# Draft Genome Sequence of Erythromycin- and Oxytetracycline-Sensitive *Nocardia seriolae* Strain U-1 (NBRC 110359)

**DOI:** 10.1128/genomeA.01606-15

**Published:** 2016-01-21

**Authors:** Masayuki Imajoh, Masaki Sukeda, Masato Shimizu, Jin Yamane, Kouhei Ohnishi, Syun-ichirou Oshima

**Affiliations:** a Department of Aquaculture, Fish Disease Laboratory, Kochi University, Nankoku, Kochi, Japan; b Research Institute of Molecular Genetics, Kochi University, Nankoku, Kochi, Japan; c Graduate School of Kuroshio Science, Kochi University, Nankoku, Kochi, Japan

## Abstract

In Japan, the emergence of macrolide- and oxytetracycline-resistant strains of *Nocardia seriolae* has previously been reported. Here, we describe the draft genome sequence of *N. seriolae* strain U-1, isolated in 2011 from a diseased yellowtail in Kagoshima Prefecture. The draft genome does not have any genes responsible for macrolide and tetracycline resistance.

## GENOME ANNOUNCEMENT

The genus *Nocardia* is a member of the family *Nocardiaceae* of the order *Corynebacteriales* within the class *Actinobacteria* (1). Over 100 species of the genus have been described as of November 2015 in the List of Prokaryotic Names with Standing in Nomenclature (2). They are aerobic, partially acid-fast, branching, Gram-positive bacilli. The causative agents of fish nocardiosis include *Nocardia asteroides*, *Nocardia seriolae*, and *Nocardia salmonicida* (3). In Japan, *N*. *seriolae* causes nocardiosis mainly in cultured yellowtail (*Seriola quinqueradiata*) and amberjack (*Seriola dumerili*) (4), which can be controlled by using sulfamonomethoxine and sulfisozole sodium (http://www.maff.go.jp/j/syouan/suisan/suisan_yobo/). On the other hand, the emergence of resistant strains to macrolide antibiotics (i.e., erythromycin, spiramycin, and kitasamycin) and oxytetracycline has previously been reported (5–7). We isolated seven *N*. *seriolae* strains in 2011, four in 2013, and five in 2014 from diseased yellowtail in Kagoshima Prefecture, All 16 strains were sensitive to oxytetracycline, but 10 of them were resistant to erythromycin (8). Here, we describe the draft genome of the two antibiotic-sensitive strain U-1. This strain has been deposited in the NITE Biological Resource Center (NBRC, Japan) under code NBRC 110359.

The genomic DNA of U-1 was extracted and purified according to our previous method (9). Genome sequencing was performed on a Roche 454-GS Junior System. The 454 reads were combined with the Illumina reads from the NCBI Sequence Read Archive under accession no. DRX020603, and assembled using GS De Novo Assembler version 2.9 software (Roche Diagnostics). The assembly consists of 346 large contigs (>500 bp) with a *N*_50_ value of 42,866 bp and largest contig size of 127,942 bp. The draft genome sequence of U-1 comprising 7,766,019 nucleotides was annotated using the Rapid Annotations using Subsystems Technology (RAST) server (10). RAST identified a total of 7,429 coding regions and 67 RNAs. Subsystem analysis in RAST predicted 58 genes involved in resistance to antibiotics and toxic compounds. The antibiotic resistance genes included 1 gene for resistance to vancomycin, 2 genes for resistance to fluoroquinolones, and 10 genes for β-lactamase, indicating that the draft genome of U-1 does not have any genes responsible for macrolide and tetracycline resistance.

### Nucleotide sequence accession numbers.

The draft genome sequence of U-1 has been deposited at DDBJ/EMBL/GenBank under the accession number BBYQ00000000. The version described in this paper is BBYQ01000000.
